# Development of UHPLC-Q-Exactive Orbitrap/MS Technique for Determination of Proanthocyanidins (PAs) Monomer Composition Content in Persimmon

**DOI:** 10.3390/plants13111440

**Published:** 2024-05-22

**Authors:** Xianyang Zhao, Da Ren, Rui Jin, Wenxing Chen, Liqing Xu, Dayong Guo, Qinglin Zhang, Zhengrong Luo

**Affiliations:** National Key Laboratory for Germplasm Innovation & Utilization of Horticultural Crops, College of Horticulture and Forestry Sciences, Huazhong Agricultural University, Wuhan 430070, China; zhaoxy@webmail.hzau.edu.cn (X.Z.); renda1124@163.com (D.R.); 18822040887@163.com (R.J.); chenwenxing@mail.hzau.edu.cn (W.C.); liqingxu@mail.hzau.edu.cn (L.X.); guoday@mail.hzau.edu.cn (D.G.)

**Keywords:** persimmon, EGCG, GCG, UHPLC-Q-Exactive Orbitrap/MS, proanthocyanidins

## Abstract

The main units of persimmon proanthocyanidins (PAs) are composed of flavan-3-ols including epigallocatechin gallate (EGCG) and gallocatechin gallate (GCG). Precise quantification of GCG is challenging due to its trace amounts in persimmon. In this study, to establish the optimal UHPLC-Q-Exactive Orbitrap/MS technique for the determination of PAs monomer composition in persimmon fruit flesh of different astringency types, mass spectrometry and chromatographic conditions were optimized. The results showed that when operating in negative ion mode, using a T3 chromatographic column (a type of C18 column with high-strength silica), acetonitrile as the organic phase, a 0.1% mobile phase acid content, and a mobile phase flow rate of 0.2 mL/min, the chromatographic peak shape and resolution of the PAs monomer composition improved. Additionally, there was no tailing phenomenon observed in the chromatographic peaks. At the same time, the intra-day and inter-day precision, stability, and recovery of the procedure were good. The relative standard deviation (RSD) of stability was less than 5%. The intra-day precision was in the range of 1.14% to 2.36%, and the inter-day precision ranged from 1.03% to 2.92%, both of which were less than 5%. The recovery rate ranged from 94.43% to 98.59% with an RSD less than 5%. The results showed that the UHPLC-Q-Exactive Orbitrap/MS technique established in this study can not only be used for the quantification of EGCG and GCG in persimmon fruit flesh but also be suitable for analyzing other PAs monomer compositions, providing robust support for the related research on persimmon PAs.

## 1. Introduction

Persimmon (*Diospyros kaki* Thunb.) fruit accumulates proanthocyanidins (PAs) in unique compartment cells called “tannin cells”. PAs are condensed with oral proteins during chewing, leading to strong astringency [1]. According to Aron and Kennedy (2008), flavan-3-ols comprise the major units of PAs. From a structural perspective, flavanols consist of a carbon backbone of C6–C3–C6, where C6 represents the aromatic rings (A and B rings), and C3 is the dihydrofuran ring (C ring) [2]. The A ring is analogous to the resorcinol moiety, while the B ring is similar to the catechol moiety. Monomers of flavan-3-ols are called catechins, and they differ in stereochemistry at C2 and C3, the presence or absence of galloyl groups, and hydroxylation on the B ring (Watrelot and Norton, 2020) [3]. Previous studies have indicated that the PAs in woody plants are primarily formed via the polymerization of catechin (C) [4]. Matsuo and Ito (1978) proposed that persimmon PAs are mainly composed of four catechin monomers, catechin (C), gallocatechin (GC), catechin gallate (CG), and gallocatechin gallate (GCG), with a composition ratio of approximately 1:2:1:2, linked by the C-4 position through C-8 [5]. Gu et al. (2008) discovered that the thiolysis products of PAs in persimmon fruit flesh consist of GC, epigallocatechin gallate (EGCG), epicatechin gallate (ECG), and an unknown monomer in a ratio of 1:7:3:1 [6]. Li et al. (2010) found that the extension units of persimmon PAs include epicatechin (EC), epigallocatechin (EGC), EGCG, and ECG, while the terminal structural units are EGCG and myricetin. In contrast to other plant PAs, persimmon PAs have a mixture of B-type and A-type connections, with A-type linkages predominating [7]. The synthesis of catechin and epicatechin units that give rise to proanthocyanidins involves the biosynthetic pathways of shikimic acid, phenylpropanoids, and flavonoids [8,9,10].

EGCG is the main component of PAs in persimmon fruit flesh, existing in the form of extension units [11]. GCG is present in trace amounts in persimmon fruit flesh. Ancillotti found that the content of GCG was the lowest compared with that of other catechin substances, measuring only 1.4 μg/100 g dw [12]. Due to the challenge of quantifying trace amounts of GCG, there are limited studies on GCG in persimmon fruit flesh. At present, HPLC is commonly used to determine the composition of PAs in persimmon fruit flesh. This method is known for its simplicity and wide applicability [13,14]. Suzuki et al. (2005) conducted qualitative and quantitative analyses of catechin substances in the fruit flesh of five persimmon varieties using HPLC. They found that the content of EGC in the astringent phenotype (non-PCNA) was higher than that in pollination-constant and non-astringent (PCNA) persimmon [15]. Veberic et al. (2010) conducted a quantitative analysis of secondary metabolites in mature fruits of 11 persimmon varieties via HPLC and found significant differences in the content of C among different varieties [16]. According to previous reports, EC, EGC, EGCG, and ECG were identified as the extension units of persimmon PAs using phloroglucinol combined with HPLC analysis [17]. The quantitative accuracy of HPLC in complex samples is limited; it is particularly challenging for trace analysis, and more suitable for samples with relatively high concentrations of the target components. GCG is the enantiomer of EGCG, and it is difficult to separate the chromatographic peaks due to their similar polarities [18,19]. Additionally, due to the low content of GCG in persimmon fruit flesh, HPLC is unable to accurately quantify it. UHPLC-Q-Exactive Orbitrap/MS enables rapid and accurate qualitative and quantitative analysis of target compounds in complex samples, providing the benefits of precise analytical results and a low detection limit [20,21,22]. However, there is no relevant study on the application of a UHPLC-Q-Exactive Orbitrap/MS procedure to determine the content of PAs monomer compositions in persimmon fruit flesh. This study aims to establish a simple, rapid, and highly sensitive UHPLC-Q-Exactive Orbitrap/MS procedure for the precise determination of monomer compositions of PAs in persimmon fruit flesh.

## 2. Materials and Methods

### 2.1. Chemicals and Reagents

Acetone, formic acid, chloroform, n-hexane, and acetonitrile were purchased from Merck Millipore (Darmstadt, Germany) and were all LC-MS-grade. Standard compounds EGCG, GCG, C, EC, EGC, ECG, and GC were purchased from Yuanye Bio-Technology (Shanghai, China). Ultrapure water, obtained from an Elga Purelab water purification system (Elga Lab Water, High Wycombe, UK), was used for determination. The EGCG, GCG, C, EC, EGC, ECG, and GC standards were accurately weighed, and LC-MS-grade methanol (Merck Millipore, Darmstadt, Germany) was used as the solvent to prepare the EGCG, GCG, C, EC, EGC, ECG, and GC standard solution with a concentration of 100 μg/mL for subsequent experiments.

### 2.2. Plant Material

Fruit samples were collected at 2.5, 10, and 27.5 weeks after bloom (WAB) from the Persimmon Repository of Huazhong Agricultural University, Wuhan, China, including different astringent types: ‘Youhou’ (PCNA), ‘Mopanshi’ (non-PCNA). All of the fruits were peeled, and the flesh from the equator section was cut and immediately frozen in liquid nitrogen. It was then stored in a −80 °C freezer until further analysis. For each astringent type, three trees were used as biological replicates with five fruits per tree (n = 15). We measured the chromatographic peak areas of the target compounds and calculated the content of EGCG and GCG in the samples based on the standard curve.

### 2.3. Instrumentation and Analytical Method

Quantitative analysis was conducted using UHPLC-Q-Exactive Orbitrap/MS (Thermo Scientific, Waltham, MA, USA), with a spray voltage of 3.0 kV, sheath gas flow of 40 μL/min, capillary temperature of 350 °C, and auxiliary gas flow of 10 μL/min. Mass spectrometry analyses were performed in a full MS-ddMS2 scan for the monitoring of ions of [M + H]^+^ and [M − H]^−^, with a mass range from 100 to 1500 *m*/*z*. The polarity of the mobile phase can be altered by adjusting the ratio of ultrapure water (A) and the organic phase (B) to achieve the separation of chromatographic peaks. In this study, the mobile phase consisted of acetonitrile (B) containing 0.1% formic acid (LC-MS grade) and ultrapure water (A) containing 0.1% formic acid (LC-MS grade). The gradient of mobile phase is shown in Table 1. Compound chromatogram peaks areas were calculated using Xcalibur 4.1 software (Thermo Scientific).

### 2.4. Extraction of PAs in Persimmon Fruit Flesh

PAs were extracted using the method of Peel and Dixon (2007) with minor modifications [23]. Firstly, 1.0 g of persimmon fruit flesh sample was ground in liquid nitrogen, extracted with 5 mL of 70% acetone (containing 0.1% ascorbic acid) for 2 h under dark conditions at 25 °C, and centrifuged at 5000× *g* for 10 min at 25 °C. Afterward, the supernatant was transferred to a new 10 mL centrifuge tube. The supernatant was mixed with 5 mL of chloroform, centrifuged at 5000× *g* for 10 min at 25 °C, and then extracted twice with chloroform and twice with n-hexane to remove residual fats (10 min, 10,000× *g*) at 25 °C. The resulting solution was centrifuged in a centrifugal device at 14,000× *g* for 10 min at 25 °C before UHPLC-Q-Exactive Orbitrap/MS analysis.

### 2.5. Optimization of the Analytical Method

#### 2.5.1. Selection of Ion Mode

There are two ion modes: positive ion mode and negative ion mode. The response intensity, chromatographic peak shape, and resolution of EGCG and GCG in the mixed standard were determined under two ion modes, and we selected the most appropriate ion mode based on the results.

#### 2.5.2. Selection of Chromatographic Column

The chromatographic column is a crucial factor that affects the resolution of target compounds. Different types of chromatographic columns are suitable for separating substances with different chemical properties, so the selection of appropriate chromatographic columns is key. In this study, the chromatographic peak shape and resolution of EGCG and GCG in the mixed standard were determined using a T3 chromatographic column (150 × 2.1 mm, 1.8 μm, 100A, ACQUITY UPLC HSS, Waters, Milford, MA, USA) and a C18 chromatographic column (100 × 2.1 mm, 3 μm, 175 A, Hypersil GOLD, Thermo Scientific, Vilnius, Lithuania). Subsequently, the optimal chromatographic column was selected.

#### 2.5.3. Selection of Mobile Phase

The mobile phase consists of ultrapure water and an organic phase. Commonly used organic phases include methanol and acetonitrile. Methanol (containing 0.1% formic acid) and acetonitrile (containing 0.1% formic acid) were chosen as the organic phases, a T3 chromatographic column was applied, and the optimal mobile phase was determined by evaluating the response intensity, chromatographic peak shape, and resolution of EGCG and GCG in the mixed standard.

#### 2.5.4. Selection of Mobile Phase Acid Content

The addition of formic acid, acetic acid, and other additives to the mobile phase can enhance the resolution efficiency of the target compound. Using ultrapure water as the aqueous phase and acetonitrile as the organic phase, formic acid was added to the mobile phase, and different acid content gradients were set to screen the optimal acid content of the mobile phase: 0.05% formic acid/mobile phase (*v*/*v*), 0.1% formic acid/mobile phase (*v*/*v*), 0.2% formic acid/mobile phase (*v*/*v*), 0.4% formic acid/mobile phase (*v*/*v*), and 0.8% formic acid/mobile phase (*v*/*v*). The T3 chromatographic column was applied. The optimal mobile phase acid content was determined based on the response intensity, chromatographic peak shape, and resolution of EGCG and GCG in the mixed standard.

#### 2.5.5. Screening of Mobile Phase Flow Rate

The flow rate of the mobile phase can affect the chromatographic peak shape, ionization efficiency, retention time (RT), and resolution of target compounds. In order to explore the optimal mobile phase flow rate, the chromatographic method was analyzed using three different mobile phase flow rates: 0.1 mL/min, 0.2 mL/min, and 0.3 mL/min. Then, the optimal mobile phase flow rate was determined by evaluating the response intensity, chromatographic peak shape, and resolution of EGCG and GCG in the mixed standard.

### 2.6. Validation of Procedure Wide Suitability

In addition to EGCG and GCG, persimmon PAs also contain other monomer compositions such as C, EC, EGC, ECG, and GC. We utilized the aforementioned procedure to identify seven monomer compositions and evaluated its suitability for the simultaneous detection of multiple persimmon PAs compositions by examining the chromatographic peak shapes and resolution of EGCG, GCG, C, EC, EGC, ECG, and GC in the mixed standard.

### 2.7. Method Validation

#### 2.7.1. Linearity

The linearity, intra-day and intra-day precision, stability, recovery, limit of detection (LOD), and limit of quantification (LOQ) were evaluated in accordance with the FDA guide [24]. The standard solution of EGCG, GCG, C, EC, EGC, ECG, and GC with a concentration of 100 μg/mL was diluted with methanol. The concentrations of EGCG, EC, ECG, and EGC were 0.1, 1, 10, 20, 50, 80, and 100 μg/mL. The concentrations of GCG were 0.01, 0.1, 2, 4, 6, 8, and 10 μg/mL. The concentrations of GC and C were 20, 15, 10, 5, 1, 0.1, and 0.05 μg/mL. Each calibration point was repeated 3 times. The standard curve was established with the concentration of the target substance plotted on the abscissa (X-axis) and the chromatographic peak areas on the ordinate (Y-axis).

#### 2.7.2. Test of Intra-Day and Inter-Day Precision

The samples (‘Mopanshi’, 2.5WAB) spiked with standard solutions of EGCG, GCG, C, EC, EGC, ECG, and GC at three different concentrations of 10, 50, and 200 ng/mL were analyzed 5 times for both intra-day and inter-day precision tests on the same day, with experiments repeated consecutively for 3 days. The chromatographic peak area of the target compound was calculated, and the intra-day and inter-day precision were present as RSDs.

#### 2.7.3. Test of Stability

The chromatographic peak area of the target compound was measured in the PAs extraction solutions from ‘Youhou’ fruit flesh. The solutions were stored at room temperature for 0, 2, 4, 8, 12, and 16 days, and the measurement was repeated three times. The RSD was calculated based on the chromatographic peak areas to evaluate the stability of EGCG, GCG, C, EC, EGC, ECG, and GC in the samples.

#### 2.7.4. Test of Recovery Rate

For the determination of the recovery rates, three content levels of the EGCG, GCG, C, EC, EGC, ECG, and GC standards were spiked to the fruit flesh sample (‘Mopanshi’, 2.5WAB) ground in liquid nitrogen before extracting PAs. Subsequently, the sample was extracted and analyzed following the procedure outlined above. Each sample was analyzed 3 times. The average recoveries were calculated using the following formula: recovery (%) = (Detected mean − Original mean)/Spiked mean × 100%.

#### 2.7.5. Limit of Detection and Quantification (LOD and LOQ)

The limit of detection (LOD) is the lowest concentration of an analyte that can be reliably distinguished from the background level during the analysis process, while the limit of quantification (LOQ) is the lowest concentration of an analyte that can be quantified. For each analyte, the LOD and LOQ are determined as concentrations with a signal-to-noise ratio of 3 and 10, respectively.

### 2.8. Statistical Analysis

Data were expressed as the mean ± standard deviation (SD) of three replicates and analyzed via analysis of variance (ANOVA) followed by Duncan’s multiple range test and *t* test, with SPSS version 17.0 (SPSS Inc., Chicago, IL, USA). Data were considered significant at *p* < 0.05.

## 3. Results and Discussion

### 3.1. Selection of Ion Mode

**Figure 1:**
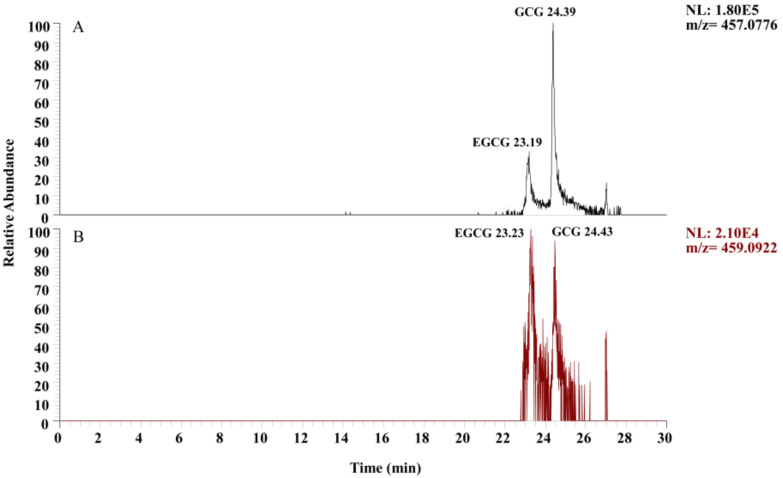
Chromatograms of EGCG and GCG in different ion modes: (**A**) negative ion mode; (**B**) positive ion mode.

Positive ion mode and negative ion mode are commonly used ion modes in mass spectrometry analysis. The choice of ion mode depends on the properties of the target compound in the sample to be analyzed. The chromatograms of EGCG and GCG in the standard solution under different ion modes are shown in Figure 1. The response intensity, chromatographic peak shape, and resolution of the target compounds were compared and analyzed in positive and negative ion modes. It was found that the ion response intensity of EGCG and GCG in negative ion mode (Figure 1A) was stronger than that in positive ion mode (Figure 1B), and the peak shape was better in negative ion mode than in positive ion mode. Therefore, the negative ion mode was used for subsequent analysis.

### 3.2. Selection of Chromatographic Column

**Figure 2:**
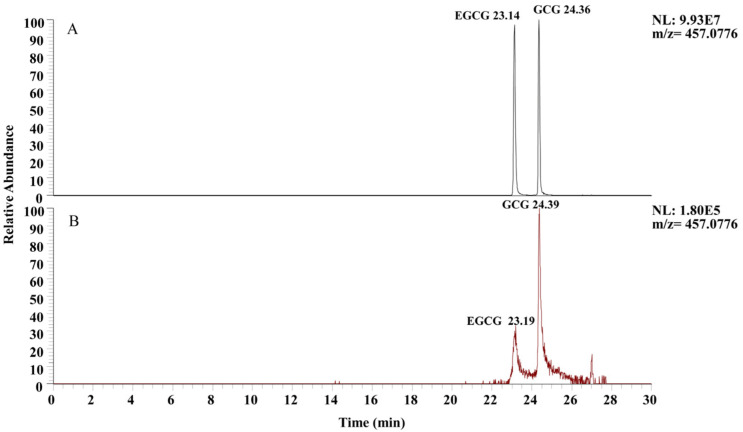
Chromatograms of EGCG and GCG with different chromatographic columns: (**A**) T3 chromatographic column; (**B**) C18 chromatographic column.

A critical aspect of LC-MS is selecting an appropriate chromatographic column. The retention time, dead time, peak height, and peak shape of the target compound were influenced by the chemical properties of the chromatographic column [25]. The T3 column is a reversed-phase C18 column based on an ultrapure silica gel matrix, which can be utilized to separate compounds with strong polarity and can maintain excellent performance over a broad pH range. The column also exhibits an excellent resolution effect on complex samples [26]. Moreover, the T3 chromatographic column is longer and has a smaller particle size, which will also affect the separation efficiency of compounds. The chromatograms of EGCG and GCG in standard solution under C18 and T3 chromatographic column conditions are shown in Figure 2. Under the conditions of the T3 chromatographic column (Figure 2A), the chromatographic peak shapes of the two target compounds were superior to those of the C18 chromatographic column (Figure 2B), the resolution degree was excellent, with no tailing observed, and the ion response intensity was better. Therefore, the T3 chromatographic column was selected for further analysis.

### 3.3. Selection of Mobile Phase

**Figure 3:**
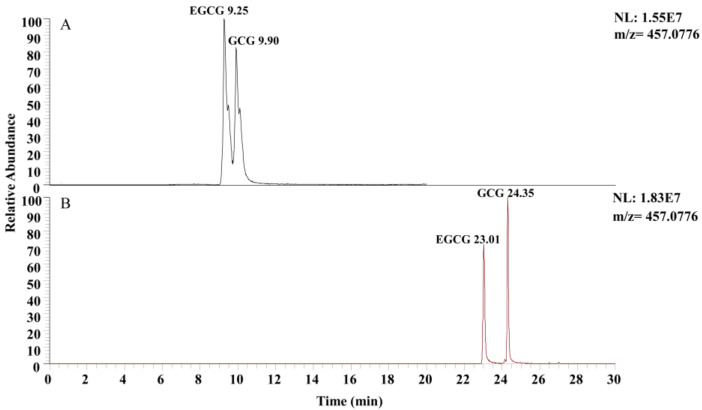
Chromatograms of EGCG and GCG of different organic phases: (**A**) methanol; (**B**) acetonitrile.

The mobile phase is a crucial factor affecting the performance of liquid chromatography. The type of organic phase affects the retention time, chromatogram peak shape, and resolution of the target compound [27]. Commonly used organic phases include methanol, acetonitrile, isopropanol, tetrahydrofuran, and other organic phases [28]. In this study, ultrapure water (containing 0.1% formic acid) was used as the aqueous phase, while methanol (containing 0.1% formic acid) and acetonitrile (containing 0.1% formic acid) were used as the organic phases for comparison (Figure 3). When methanol was used as the organic phase (Figure 3A), the retention time of the target compounds was shorter. However, the chromatographic peaks of EGCG and GCG were not completely separated. When acetonitrile was used as the organic phase (Figure 3B), the chromatographic peaks of the target compounds exhibited good shapes and showed better resolution, indicating that acetonitrile yielded superior results as the organic phase.

### 3.4. Selection of Mobile Phase Acid Content

**Figure 4:**
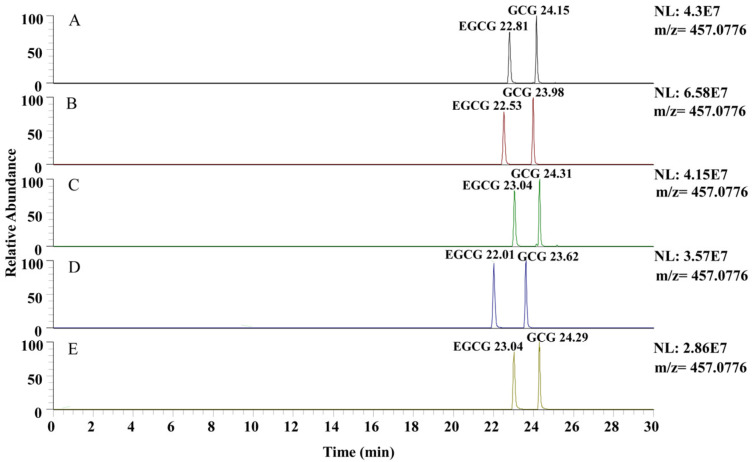
Chromatograms of EGCG and GCG of different mobile phase acid contents: (**A**) 0.05%; (**B**) 0.1%; (**C**) 0.2%; (**D**) 0.4%; (**E**) 0.8%.

**Figure 5:**
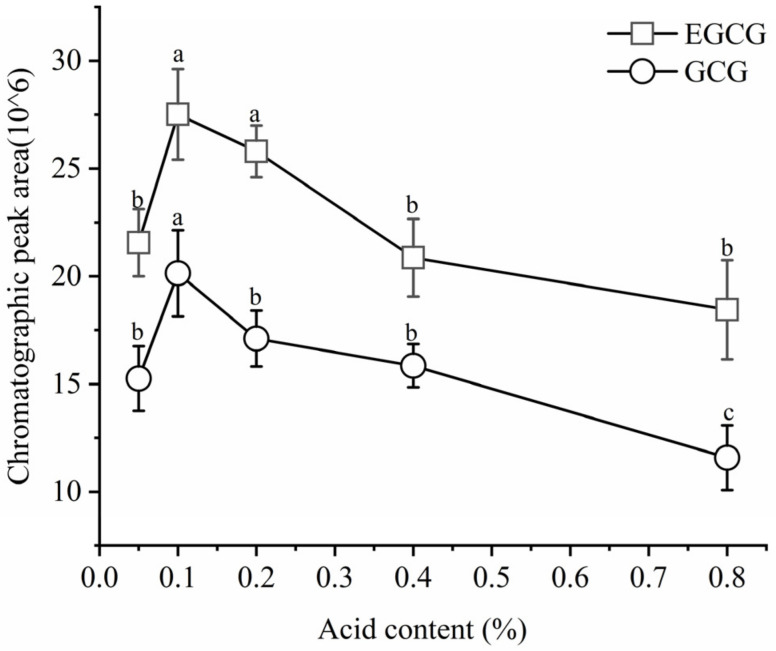
Chromatographic peak area of EGCG and GCG in different mobile phase acid contents. Different letters indicate significant differences between different acid contents at the *p* < 0.05 level. The chromatographic peak area represented by “a” is significantly higher than that represented by “b” and “c”, and the peak area represented by “b” is significantly higher than that represented “c”.

The addition of additives to the mobile phase can affect the ion response intensity, chromatographic peak resolution, and retention time. Common mobile phase additives include formic acid, acetic acid, and trifluoroacetic acid [29]. In this experiment, different mobile phase acid contents (*v*/*v*) were utilized to analyze the standard solutions of EGCG and GCG. As shown in Figure 4, the range of mobile-phase acid contents was from 0.05% to 0.8%, and the target compounds exhibited good resolution, with no significant differences observed. However, the ion response intensity was different. Based on the chromatographic peak areas for the relative quantification of EGCG and GCG (Figure 5), it was observed that the chromatographic peak areas exhibited an increasing trend followed by a decrease within the acid content range of 0.05% to 0.8%. When the acid content was 0.1%, the chromatographic peak area of EGCG and GCG was the highest, significantly higher than that at other acid contents. This suggested that the optimal effect is achieved when the acid content in the mobile phase is 0.1% (Figure 5).

### 3.5. Screening of Mobile Phase Flow Rate

**Figure 6:**
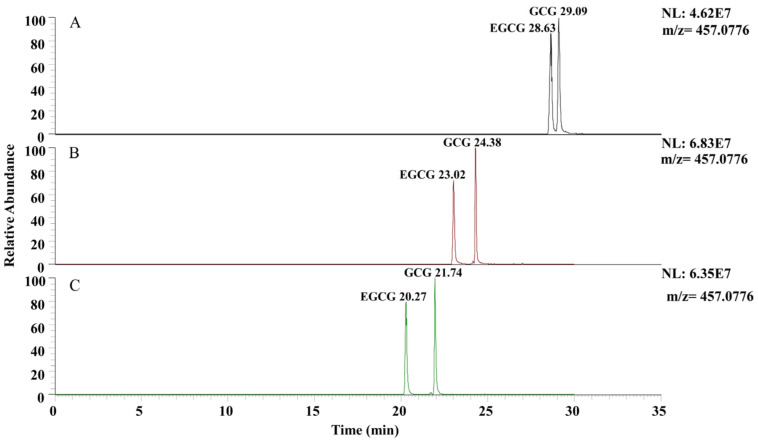
Chromatograms of EGCG and GCG at different mobile phase flow rates. (**A**) 0.1 mL/min; (**B**) 0.2 mL/min; (**C**) 0.3 mL/min.

Related studies indicate that increasing the flow rate of the mobile phase can enhance the chromatographic peak resolution of target compounds [30]. As shown in Figure 6, a comparative analysis of different mobile phase flow rates revealed that at a flow rate of 0.1 mL/min, there was a delay in the retention times, leading to poorer resolution. At flow rates of 0.2 mL/min and 0.3 mL/min, the target compounds exhibited better separation, with ion response intensities superior to those at 0.1 mL/min. Additionally, there were no significant differences in the response intensity and chromatographic peak shapes between these two flow rates. When the flow rate was 0.2 mL/min, the column pressure was lower than when the flow rate was 0.3 mL/min. Lower column pressure extends the lifespan of the chromatographic column. Therefore, a flow rate of 0.2 mL/min was considered optimal for the mobile phase.

### 3.6. Validation of Procedure Wide Suitability

**Figure 7:**
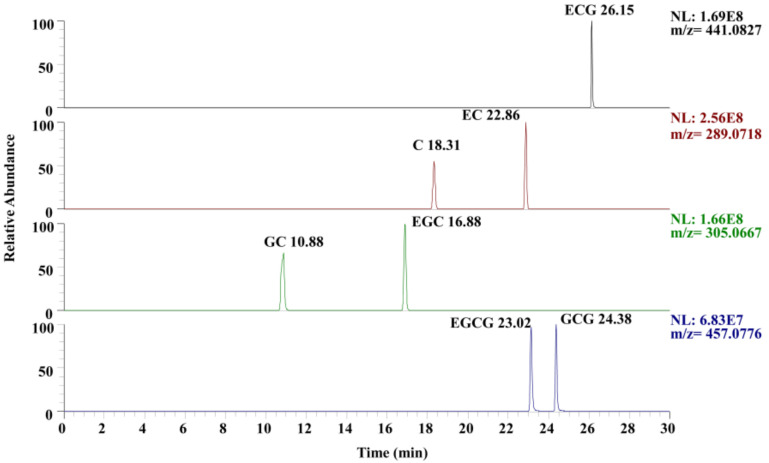
Chromatograms of PAs monomer compositions.

The chromatograms of the seven monomer compositions of persimmon PAs are presented in Figure 7. Based on the UHPLC-Q-Exactive Orbitrap/MS procedure developed in this study, the chromatographic peaks of each target compound exhibited excellent peak shapes without tailing phenomena. Moreover, there was good separation between isomers (EGCG and CGG, C and EC, and GC and EGC), indicating that this procedure can not only determine EGCG and GCG but also detect other monomer compositions.

### 3.7. Linearity

The linearities of PAs monomer compositions are shown in Table 2. All compositions exhibited good linearity within different concentration ranges, with R^2^ ranging from 0.9984 to 0.9998. The regression equation of EGCG was y = 831,335.87x + 100,728.46, with R^2^ = 0.9991, suggesting good linearity between the chromatographic peak area and EGCG concentration within the range of 0.1–100 μg/mL. The regression equation of GCG was y = 2,272,957.25x – 26,980.21, and R^2^ = 0.9998. The results showed that the chromatographic peak area and concentration of GCG showed good linearity in the range of 0.01–10 μg/mL.

### 3.8. Test of Intra-Day and Intra-Day Precision

As shown in Table 3, the intra-day precision for PAs monomer compositions was in the range of 1.14% to 2.36% (n = 5). Further, the inter-day precision of this method for PAs monomer compositions ranged from 1.03% to 2.92%, which is within the acceptance criteria of the FDA guide for validating analytical methods. This indicated that the experimental instrument and analysis method have good precision and can be applied to sample analysis.

### 3.9. Test of Stability

Table 4 shows the stability of PAs monomer compositions in the sample after storage at room temperature for 16 days. The RSD of the chromatographic peak areas of PAs monomer compositions were distributed between 1.08% and 1.98%, indicating that the stability of them was good after 16 days of storage at room temperature.

### 3.10. Test of Recovery

The results of recovery are shown in Table 5. The recovery of PAs monomer compositions ranged from 94.43% to 98.59%, and the RSD ranged from 0.48% to 4.78%. This indicates excellent recovery for all monomer compositions based on the proposed method.

### 3.11. Limit of Detection and Quantification (LOD and LOQ)

As displayed in Table 6, the LOD and LOQ for PAs monomer compositions were determined at an S/N of approximately 3 and 10, respectively. LOD values ranged from 0.000077 μg/mL to 0.00039 μg/mL, and LOQ values ranged from 0.00029 μg/mL to 0.00129 μg/mL.

### 3.12. Procedure Applicability

**Figure 8:**
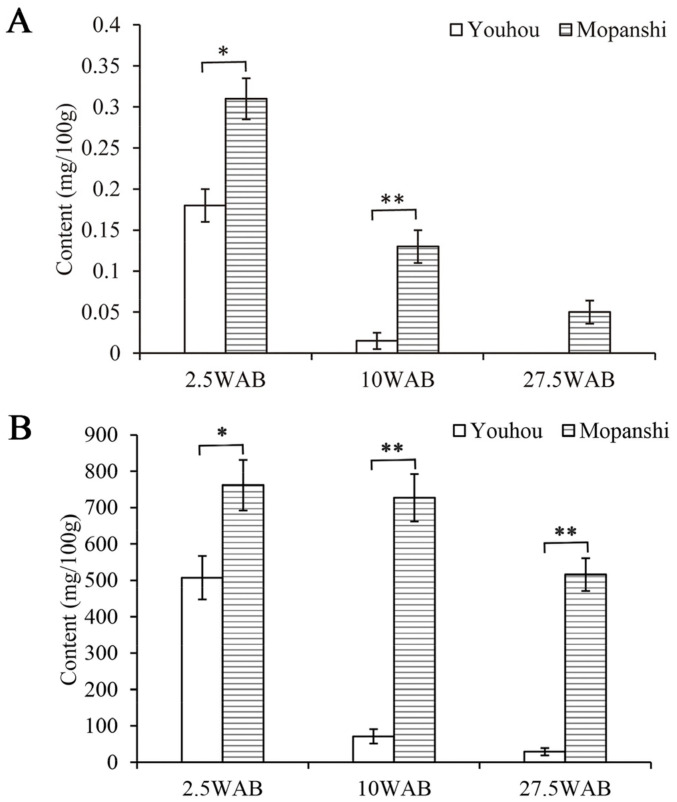
GCG (**A**) and EGCG (**B**) contents of ‘Youhou’ and ‘Mopanshi’ at different developmental stages. Note: the mixed sample of five fruits from each tree is considered one biological replicate. The data represent the averages of three biological replicates. * Significant difference at the *p* < 0.05 level; ** significant difference at the *p* < 0.01 level.

Based on the UHPLC-Q-Exactive Orbitrap/MS procedure established in this study, the EGCG and GCG content of PAs in the fruit flesh of ‘Youhou’ (PCNA) and ‘Mopanshi’ (non-PCNA) at different developmental stages (2.5, 10, and 27.5 WAB) was determined. The results are shown in Figure 8. The GCG content of PAs in the fruit flesh of ‘Mopanshi’ was significantly higher than that in ‘Youhou’ at 2.5 and 10 WAB. As fruit development progressed, only trace amounts of GCG were detected in ‘Mopanshi’ at 27.5 WAB (Figure 8A). In comparison with GCG, both PCNA and non-PCNA exhibited higher levels of the EGCG content of PAs in their fruit flesh. Throughout the entire developmental period, the EGCG content in non-PCNA fruit flesh was significantly higher than that in PCNA fruit flesh (Figure 8B). This conclusion was consistent with that of previous studies of Akigi: the concentration of the main PAs component in persimmon fruit, EGCG, varies significantly among astringency types, with the EGCG content of non-PCNA being higher than that of PCNA. However, the presence of GCG was not detected in his research [11], and combined with the methodological investigation in this study, this indicates that the analysis method can not only be used for the quantification of EGCG and GCG in persimmon fruit flesh but also be suitable for analyzing other PAs monomer compositions. In this study, a method was established for determining the content of PAs monomer compositions in persimmon fruit flesh using UHPLC-Q-Exactive Orbitrap/MS. Compared with traditional HPLC analysis, the method used in this study exhibited robust separation capability, high sensitivity, and reliable qualitative analysis results, providing a valuable reference for further research on PAs monomer compositions in persimmon fruit flesh.

## 4. Conclusions

In this study, a UHPLC-Q-Exactive Orbitrap/MS procedure was developed for the precise quantification of PAs monomer compositions in persimmon fruit flesh. This was established by optimizing the chromatographic column type, ion mode, mobile phase type, mobile phase acid content, and flow rate of the mobile phase. It was found that when operating in negative ion mode, using a T3 chromatographic column, acetonitrile as the organic phase, a 0.1% mobile phase acid content, and a mobile phase flow rate of 0.2 mL/min, the chromatographic peaks of EGCG and GCG showed excellent peak shapes and separation efficiency, with no tailing phenomenon observed. Meanwhile, based on this procedure, in addition to GCGC and GCG, the chromatographic peaks of C, EC, GC, EGC, and ECG also exhibited good peak shapes without tailing phenomena and excellent separation between isomers (EGCG and CGG, C and EC, GC, and EGC). The PAs monomer compositions exhibited good linearity within different concentration ranges. The intra-day and inter-day precision, stability, and recovery of the procedure were satisfactory. The recovery rate of PAs monomer compositions was 94.43% to 98.59%, while the intra-day precision was in the range of 1.14% to 2.36% (n = 5), and the inter-day precision was 1.03–2.92%. The above conclusions indicate that the procedure could not only be used to quantify the content of EGCG and GCG but also be suitable for the analysis of several other monomer compositions. This could provide strong support for the related research on persimmon PAs.

## Figures and Tables

**Table 1:** Gradient of mobile phase.

Time (min)	Volume Fraction of Flow Phase (%)	
	A	B
0	98	2
5	95	5
15	90	10
20	85	15
24	70	30
25	0	100
27	0	100
27.1	98	2
30	98	2

**Table 2:** Linearity for PAs monomer compositions.

Compound	Regression Equation	R^2^	Range of Linear μg/mL
EGCG	y = 831,335.87x + 100728.46	0.9991	0.1–100
GCG	y = 2,272,957.25x − 26980.21	0.9998	0.01–10
C	y = 2,412,557.34x − 307572.82	0.9993	0.05–20
EC	y = 756,198.76x − 393787.58	0.9994	0.1–100
ECG	y = 647,426.35x − 392818.31	0.9986	0.1–100
GC	y = 2,821,093.03x − 276398.98	0.9984	0.05–20
EGC	y = 520,917.04x − 269596.49	0.9992	0.1–100

Note: The data in the table are averages of 3 replicates.

**Table 3:** Intra-day and inter-day precision of PAs monomer compositions.

Compound	Spiked Amount/μg	Intra-Day Precision (RSD)	Inter-Day Precision (RSD)
EGCG	200.00	1.60	1.75
	50.00	1.59	1.83
	10.00	1.61	2.53
GCG	200.00	1.32	1.72
	50.00	1.36	2.23
	10.00	1.14	1.29
C	200.00	1.83	1.45
	50.00	2.11	2.92
	10.00	1.38	1.92
EC	200.00	1.52	1.12
	50.00	2.36	1.17
	10.00	1.65	2.07
GC	200.00	2.12	2.71
	50.00	1.39	1.34
	10.00	1.87	2.29
EGC	200.00	1.32	1.12
	50.00	1.18	1.23
	10.00	1.73	2.81
ECG	200.00	2.02	1.14
	50.00	1.86	1.03
	10.00	2.35	2.32

Note: The data in the table are the averages of 5 replicates.

**Table 4:** Stability of PAs monomer compositions in sample.

Compound	Peak Area					Average	RSD/%
	0 d	2 d	4 d	8 d	16 d		
EGCG	66,709,150.2	68,352,315.15	66,328,946.25	66,731,925.7	65,704,110.4	66,765,289.6	1.47
GCG	55,989.15	57,542.1	55,828.5	57,850.8	57,437.1	56,929.5	1.66
C	4,414,029.3	4,412,461.3	4,395,743	4,342,236.6	4,364,036.3	4,385,701.3	1.52
EC	131,509,817	136,223,418.3	133,189,066	131,890,391	131,018,021.7	132,766,142.8	1.98
GC	4,064,545	4,065,587.3	4,102,328.3	4,071,499.7	4,091,376.7	4,079,067.4	1.29
EGC	162,794,473	160,009,289.7	160,713,249.3	161,670,081.3	161,843,229.3	161,406,064.5	1.21
ECG	60,516,798.7	61,145,932.7	60,832,165	60,969,459.7	61,332,962	60,959,463.6	1.08

Note: The data in the table are the averages of 3 replicates.

**Table 5:** Recovery of PAs monomer compositions.

Compound	Spiked Mean/mg	Recovery/%	RSD/%
EGCG	5	96.89	1.25
	2	95.25	1.66
	1	98.59	0.48
EC	5	95.70	1.47
	2	94.52	2.47
	1	96.32	3.70
EGC	5	97.64	1.01
	2	95.66	2.96
	1	96.42	0.61
ECG	5	96.77	1.18
	2	94.96	2.30
	1	96.09	2.32
GCG	0.05	96.67	4.78
	0.03	94.43	2.04
	0.02	97.45	1.08
C	0.05	96.10	2.33
	0.03	95.97	1.25
	0.02	94.67	1.99
GC	0.05	95.58	1.05
	0.03	95.30	2.35
	0.02	96.27	2.87

Note: The data in the table are the averages of 3 replicates.

**Table 6:** LOD and LOQ for PAs monomer compositions.

Compound	LOD (μg/mL)	LOQ (μg/mL)
EGCG	0.00031	0.00103
GCG	0.00028	0.00094
C	0.00014	0.00046
EC	0.000077	0.00026
ECG	0.00017	0.00056
GC	0.00039	0.00129
EGC	0.000086	0.00029

Note: The data in the table are the averages of 3 replicates.

## Data Availability

Data are contained within the article.
